# Heart Failure and Laparoscopic Cholecystectomy for Acute Cholecystitis: The Need for Frailty Assessment, Biomarkers, and MINS Surveillance

**DOI:** 10.14309/ctg.0000000000000984

**Published:** 2026-02-23

**Authors:** Shameer Tahir

**Affiliations:** 1 Allama Iqbal Medical College, Lahore, Pakistan.

I read with great interest the study by Pai and Hsieh evaluating the impact of pre-existing heart failure (HF) on outcomes after laparoscopic cholecystectomy for acute cholecystitis (1). This large-scale analysis addresses an important clinical question, and the authors are commended for highlighting a vulnerable subgroup within emergency biliary surgery. Nevertheless, several considerations may enhance interpretation and translational relevance.

First, although propensity-score matching was used to reduce confounding, the analysis does not fully address the role of frailty, an increasingly recognized determinant of postoperative adverse cardiovascular events. Frailty frequently coexists with HF and independently predicts myocardial infarction, cardiac arrest, and mortality after noncardiac surgery (2). Adjusting for frailty may help distinguish the risk attributable directly to HF from that related to global physiological reserve.

Second, the markedly increased incidence of perioperative myocardial infarction and cardiopulmonary complications among patients with HF should be interpreted in the context of contemporary understanding of perioperative myocardial injury (MINS). MINS is often clinically silent yet strongly prognostic, and its detection relies on routine postoperative troponin measurement—an element absent from the present dataset. Recent studies emphasize that subclinical myocardial injury is common after noncardiac surgery and associated with short-term and long-term mortality (3). Furthermore, the 2024 American Heart Association/American College of Cardiology perioperative cardiovascular guidelines recommend postoperative troponin surveillance in high-risk surgical patients, including those with known cardiovascular disease or aged 65 years or older (4). Without accounting for MINS, the study may underestimate the true cardiovascular burden in patients with HF.

Third, the analysis does not incorporate preoperative cardiac biomarkers, such as NT-proBNP, which have demonstrated greater predictive accuracy for postoperative morbidity and may better stratify risk in HF populations. Prospective data show that NT-proBNP complements or outperforms conventional clinical risk indices and enhances perioperative risk discrimination (5). Incorporating such biomarkers into future work may allow more precise identification of patients with HF who would benefit from enhanced preoperative optimization or postoperative monitoring.

Finally, while Pai and Hsieh appropriately highlight the increased health care utilization associated with HF, the broader system-level implications merit further attention. Structured perioperative pathways have recently been shown to reduce mortality and unnecessary percutaneous drainage in high-risk acute cholecystitis through standardized evaluation and multidisciplinary review (6). Embedding HF-specific optimization—such as cardiology consultation, guideline-directed therapy review, and postoperative troponin surveillance—within such pathways may attenuate the excess morbidity and hospital costs identified in this study.

In summary, this important study underscores the heightened perioperative risk among patients with HF requiring cholecystectomy. Future analyses incorporating frailty assessment, biomarker-enhanced risk stratification, systematic MINS detection, and pathway-based perioperative care could improve the precision and actionable value of research in this area and better guide clinical practice.

## CONFLICTS OF INTEREST

**Guarantor of the article:** Shameer Tahir, MBBS Candidate.

**Specific author contributions:** S.T.: contributed solely to the conception, drafting, and revision of this Letter to the Editor.

**Financial support:** None to report.

**Potential competing interests:** None to report.
